# XPNPEP2 is associated with lymph node metastasis in prostate cancer patients

**DOI:** 10.1038/s41598-019-45245-5

**Published:** 2019-07-11

**Authors:** Fei Li, Yun Dai, Hao Xu, Kecheng Huang, Ying Zhou, Danfeng Luo, Ding Ma, Ling Xi, Mengqin Lv, Xiangyi Ma

**Affiliations:** 1 Department of Obstetrics and Gynecology, Tongji Hospital, Tongji Medical College, Huazhong University of Science and Technology, 1095 Jiefang Avenue, Wuhan, Hubei 430030 People’s Republic of China; 2 Department of Urology, Tongji Hospital, Tongji Medical College, Huazhong University of Science and Technology, Wuhan, Hubei 430030 China; 3 Institute of Urology, Tongji Hospital, Tongji Medical College, Huazhong University of Science and Technology, Wuhan, Hubei 430030 China; 4 Jiangxi Maternal and Child Health Hospital, 318 Bayi Avenue, Nanchang, Jiangxi 330006 China

**Keywords:** Oncogenes, Tumour biomarkers

## Abstract

As we reported in our previous studies, TMTP1, a tumor-homing peptide, selectively targets highly metastatic tumors and their metastatic foci. Aminopeptidase P2 (XPNPEP2) is a receptor for TMTP1 tumor-homing peptide. However, the biological and clinical significance of Aminopeptidase P2 in human cancers remains unknown. In this study, the high-density multiple organ tumor tissue array was employed for the analysis of XPNPEP2 expression profiles in human specimens. The results showed that XPNPEP2 was moderately expressed in the normal prostate tissues, but significantly decreased in the prostate cancer. Hence we used TCGA, IHC, and ELISA to further analyze the expression of XPNPEP2 in tissues and serum of prostate cancer patients. In general, XPNPEP2 expression was lower in prostate cancer tissue than in normal prostate tissue, but was higher in prostate cancer tissues with local invasion and LN metastasis than in tissues with localized Pca. Western blot clarified XPNPEP2 had a secreted form in the serum. Then the serums of 128 Pca patients, 70 healthy males and 40 prostate hyperplasia patients were obtained for detecting serum XPNPEP2 levels.The results indicated that the concentration of XPNPEP2 in serums of Pca patients with LN metastasis (142.7 ± 14.40 ng/mL) were significantly higher than levels in Pca patients without LN metastasis (61.63 ± 5.50 ng/mL) (*p* < 0.01). An ROC analysis revealed that the combination of PSA and XPNPEP2 was more efficient than PSA or XPNPEP2 alone for predicting LN metastasis, especially for Pca patients with low serum PSA levels. In summary, serum XPNPEP2 levels when combined with PSA levels may result in increased sensitivity for predicting LN metastasis in Pca patients, especially for patients with low serum PSA levels.

## Introduction

Prostate cancer (Pca) is the most frequent malignancy and the second leading cause of cancer-related death for males in the United States. Historically, the incidence of prostate cancer in China was lower than in the USA, however current data indicated that the occurrence of Pca was increasing^1^. Currently, prostate-specific antigen (PSA) blood test is used to screen for Pca and the condition is then diagnosed with a prostate biopsy. Although PSA is considered as the most powerful biomarker for the detection and risk classification of prostate cancer, it has noteworthy limitations^2^. In Pca patients with Gleason grade 8-10, a proportion of tumor cells are so poorly differentiated that they produce relatively little PSA^3,4^. Therefore, more precise indicators of disease severity are critical for optimizing the treatment of Pca patients and may offer predictive and prognostic clinical information^5,6^.

In our previous study, a tumor-homing peptide, TMTP1, selectively targets to the highly metastatic tumors and its metastatic foci. Aminopeptidase P2 (XPNPEP2) was recognized as a receptor for TMTP1^7–10^. However, the biological and clinical significance of Aminopeptidase P2 in human cancers remains unknown. Previously, we found that Aminopeptidase P2 was moderately expressed in prostate tissues. XPNPEP2 is an aminoacylproline hydrolase that specifically removes the N-terminal amino acid from peptides with a penultimate prolyl residue^11^. A large number of biologically active polypeptides, including hormones, growth factors, neurotransmitters, coagulating proteins, toxins, and cytokines, are potential substrates of XPNPEP2^12,13^. Importantly, XPNPEP2 acts synergistically with kallikrein during bradykinin production. PSA, i.e, human kallikrein 3 (hK3), is also a serine protease in the kallikrein family of proteases^14^, which is able to produce kinins via its kininogenase activity. Both a variant XPNPEP2 and an abnormality in the kallikrein-kinin system are associated with bradykinin-mediated angioedema^15^. A correlation between polymorphisms and haplotype mutations of XPNPEP2 and ACEI-induced angioedema has been reported^16^. However, there was little information about the physiological roles and clinical significance of XPNPEP2 in prostate diseases. In this study, we investigated the expression of XPNPEP2 in normal and cancerous prostate tissues. Meanwhile, the serum levels of XPNPEP2 in Pca patients were measured to evaluate the association between serum XPNPEP2 levels and patient clinical and pathologic characteristics, including the PSA value, pathologic Gleason score, extracapsular extension, seminal vesicle invasion and lymph node metastasis.

## Results

### Clinical characteristics

In this study, we collected serum samples from 128 prostate cancer patients with localized Pca or local invasion and LN metastasis, and two of which were lack of complete lymph nodes dissection. The median age of these Pca patients was 67 years (ranging from 51-82 years). In the benign prostate hyperplasia (BPH) group, the median age was 72 years. Clinical characteristics of human population in normal, BPH and PCa cohorts were summarized in Table 1, and clinical detains of PCa were listed in Table 2.

**Table 1:** Clinical characteristics of human populations in normal, BPH and PCa cohorts

characteristics	Normal	BPH	PCa
No. of patients	70	40	126
Age(years) Median(range)	70(50-81)	72(48-82)	67(51-82)
Serum PSA(ng/mL) Median ± SD	1.534 ± 0.988	9.236 ± 2.357	142.9 ± 21.71
Serum XPNPEP2(ng/mL) Median ± SD	85.82 ± 8.16	76.94 ± 12.86	77.35 ± 7.72

**Table 2:** Relationship between serum protein levels (XPNPEP2 and PSA) and clinicopathological variables.

	No(%)	XPNPEP2(ng/mL)	P Value	PSA(ng/mL)	P Value
**Age, yr**					
≤65	42(32.5)	87.06 ± 13.85		47.34 ± 9.08	
>65	86(67.5)	71.88 ± 10.23	P = 0.58	242.5 ± 36.10	P < 0.01*
**Biopsy Gleason Score**					
≤7	53(42)	78.54 ± 13.48		125.3 ± 39.07	
>7	73(58)	62.10 ± 8.55	P = 0.49	176.9 ± 48.86	P = 0.53
**lymph node metastasis**					
No	87(70)	61.63 ± 5.50		78.12 ± 14.59	
Yes	39(30)	142.7 ± 14.40	P < 0.01	250.8 ± 55.08	P < 0.01
**Local invasion**					
No	62(49)	77.58 ± 9.80		71.62 ± 19.90	
Yes	64(51)	95.91 ± 9.70	P = 0.19	198.0 ± 36.74	P < 0.01

### Expression of XPNPEP2 in normal tissues

Immunohistochemistry (IHC) analysis was performed to investigate the location and intensity of XPNPEP2 protein expression. XPNPEP2 protein in all organs was detected by IHC using the normal tissue microarray (MC5003a), which includes kidney, lung, liver, colon, testis, breast, prostate and other organs. XPNPEP2 protein expression was mainly observed in the membrane, the intensity of XPNPEP2 was preferentially expressed in the renal proximal tubule. Importantly, we observed that the glandular cells in the prostate had moderately high levels of XPNPEP2 protein. Moreover, the expression levels of XPNPEP2 in pancreatic islet cells, brush border membranes of intestinal epithelial cells and liver cells were weakly positive. Inversely, XPNPEP2 scarcely expressed in tissues of cervix, ovary, breast and lung. Representative immunohistochemically stained sections were shown in Supplementary Fig. 1.

### Expression of XPNPEP2 in Pca patients

**Figure 1:**
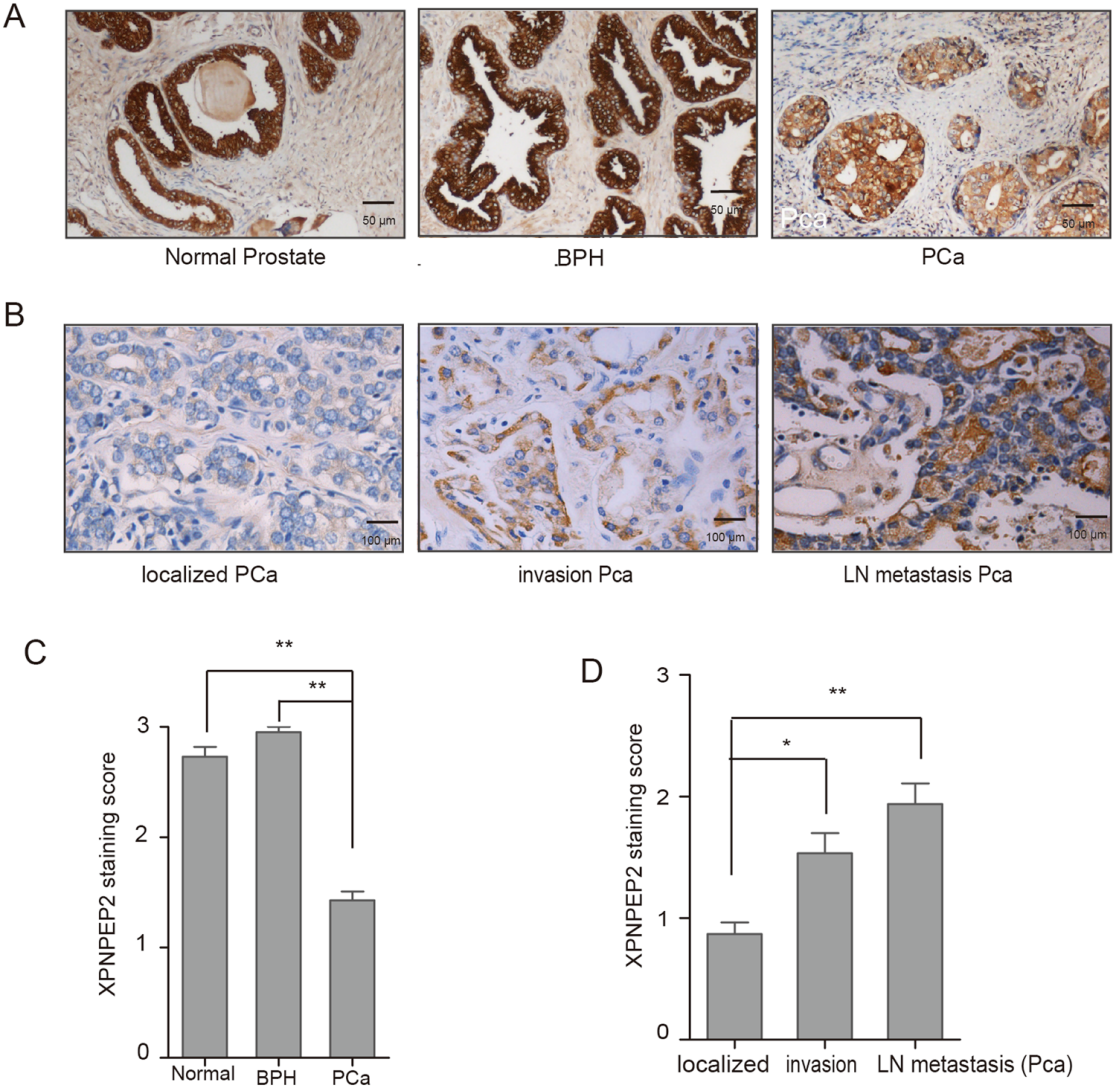
Immunostaining of XPNPEP2 expression in human prostate tissue. (**A**) and (**C**) A prostate cancer tissue microarray PR1921 and 30 BPH tissues were employed for staining with anti-XPNPEP2, the representative figures were shown (**A**), and XPNPEP2 immunostaining scores was presented (**C**). Scale bar, 50 um. **p < 0.01. (**B**) and (**D**) XPNPEP2 expression in Pca subdivided into localized, locally invasive and LN-metastatic Pca was also analyzed (**B, D**). Scale bar, 100 um. *p < 0.05, **p < 0.01.

For primary evaluation of XPNPEP2 expression in Pca patients, a prostate cancer tissue microarray and other BPH tissue samples obtained from Tongji hospital were utilized. XPNPEP2 expression was lower in prostate samples from Pca patients including localized and lymph node metastasis than normal or BPH patients (Fig. 1A). More specifically, XPNPEP2 immunostaining scores were significantly decreased in the Pca tissues (*p* < 0.01, Fig. 1C). The mRNA levels of XPNPEP2 were also reduced in PCa by GEPIA analysis (Supplementary Fig. 2).

**Figure 2:**
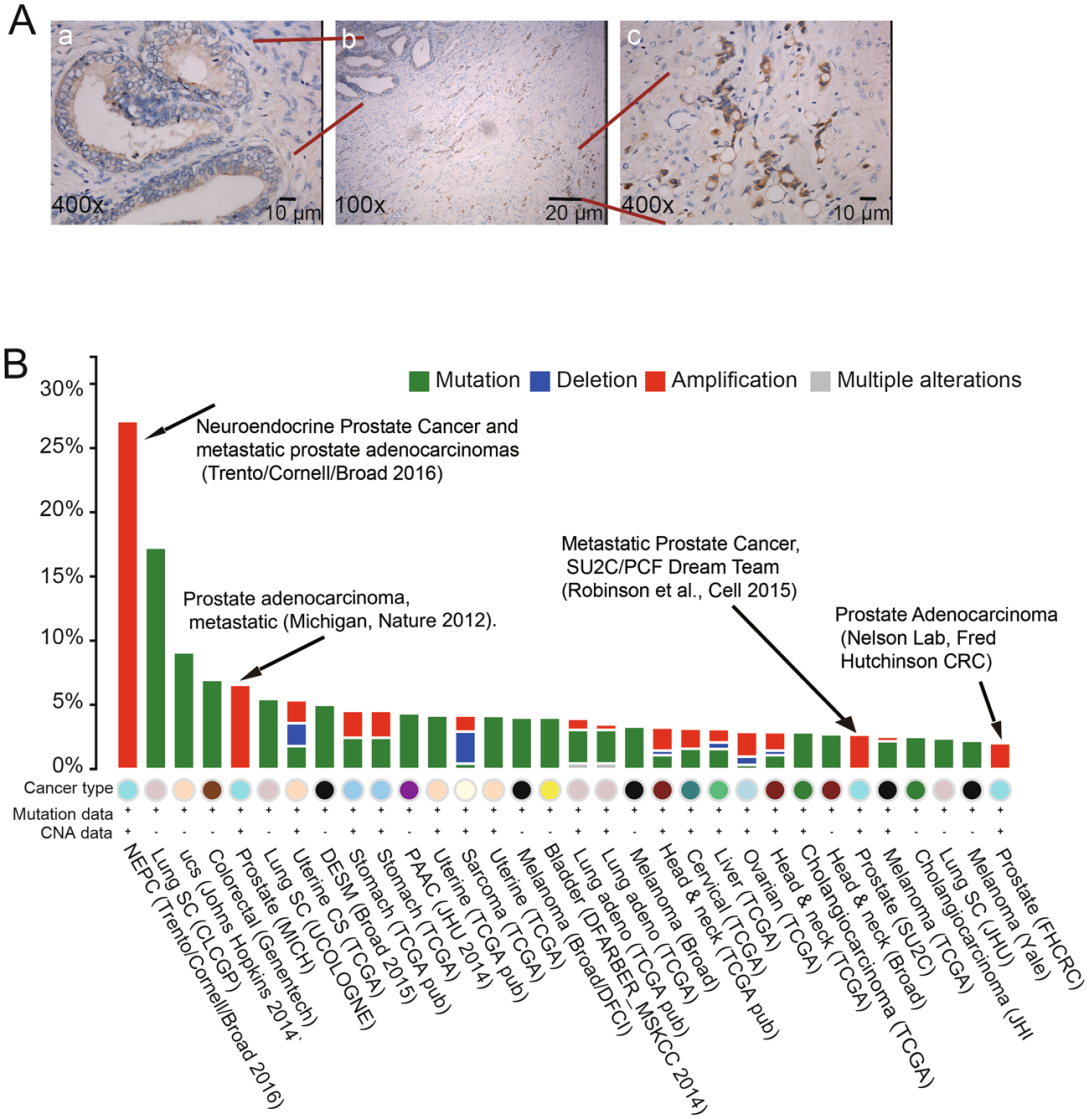
The correlation between XPNPEP2 and metastasis. In the same FFPE section, the XPNPEP2 expression in the occult metastases and in large foci were analyzed (**A**), scale bar, 10 um. The cross-cancer XPNPEP2 mutation analysis was demonstrated using data extracted from the cBioPortal online analysis tool (the cBioPortal for Cancer Genomics) (**B**).

For the evaluation of the associations between protein expression and clinicopathologic datas, XPNPEP2 expression in patients with localized, locally invasive and LN-metastatic Pca were analyzed, respectively. We found that XPNPEP2 protein was higher in locally invasive and LN-metastatic Pca than in localized Pca (*p* < 0.01, Fig. 1D). A schematic diagram of immunoreactivity scoring (IRS) was presented in Fig. 1B. In the meantime, we also demonstrated that XPNPEP2 expression in occult metastases was higher than in large foci (Fig. 2A).

To confirm whether XPNPEP2 plays a role in human prostate cancer, we analyzed XPNPEP2 gene alterations using data extracted from the cBioPortal online tool (the cBioPortal for Cancer Genomics)^17,18^. Cross-cancer XPNPEP2 alteration analysis demonstrated that XPNPEP2 is frequently altered in different types of cancers (Fig. 2B). Interestingly, among these 36 examined cancer types or subtypes, prostate cancer had the highest frequency of XPNPEP2 gene amplifications. Moreover, all studies of Pca patients were associated with metastatic prostate cancer.

### Serum XPNPEP2 protein levels in Pca patients

**Figure 3:**
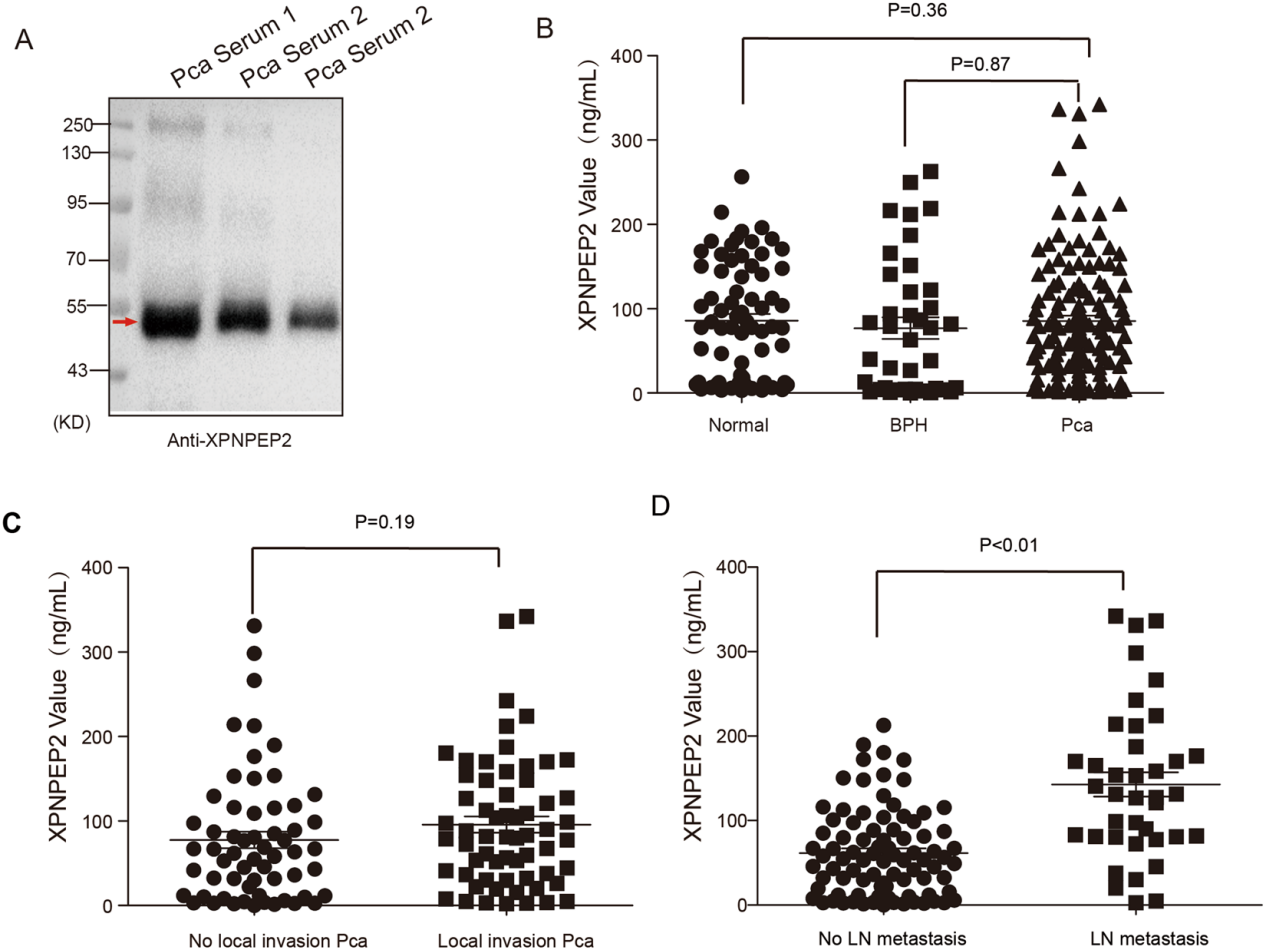
Serum XPNPEP2 levels in normal and prostate cancer patients. Western blot assay clarified secreted XPNPEP2 in serums of Pca patients (**A**). The ELISA assay detected serum XPNPEP2 levels in normal, BPH and Pca patients using a human XPNPEP2 ELISA pair set, the scatter plots were showed (**B**). (**C**) Plots illustrated the serum XPNPEP2 levels in Pca patients with local invasion versus without local invasion. (**D**) Plots showing the serum XPNPEP2 levels in Pca patients with LN metastasis versus without LN metastasis.

**Figure 4:**
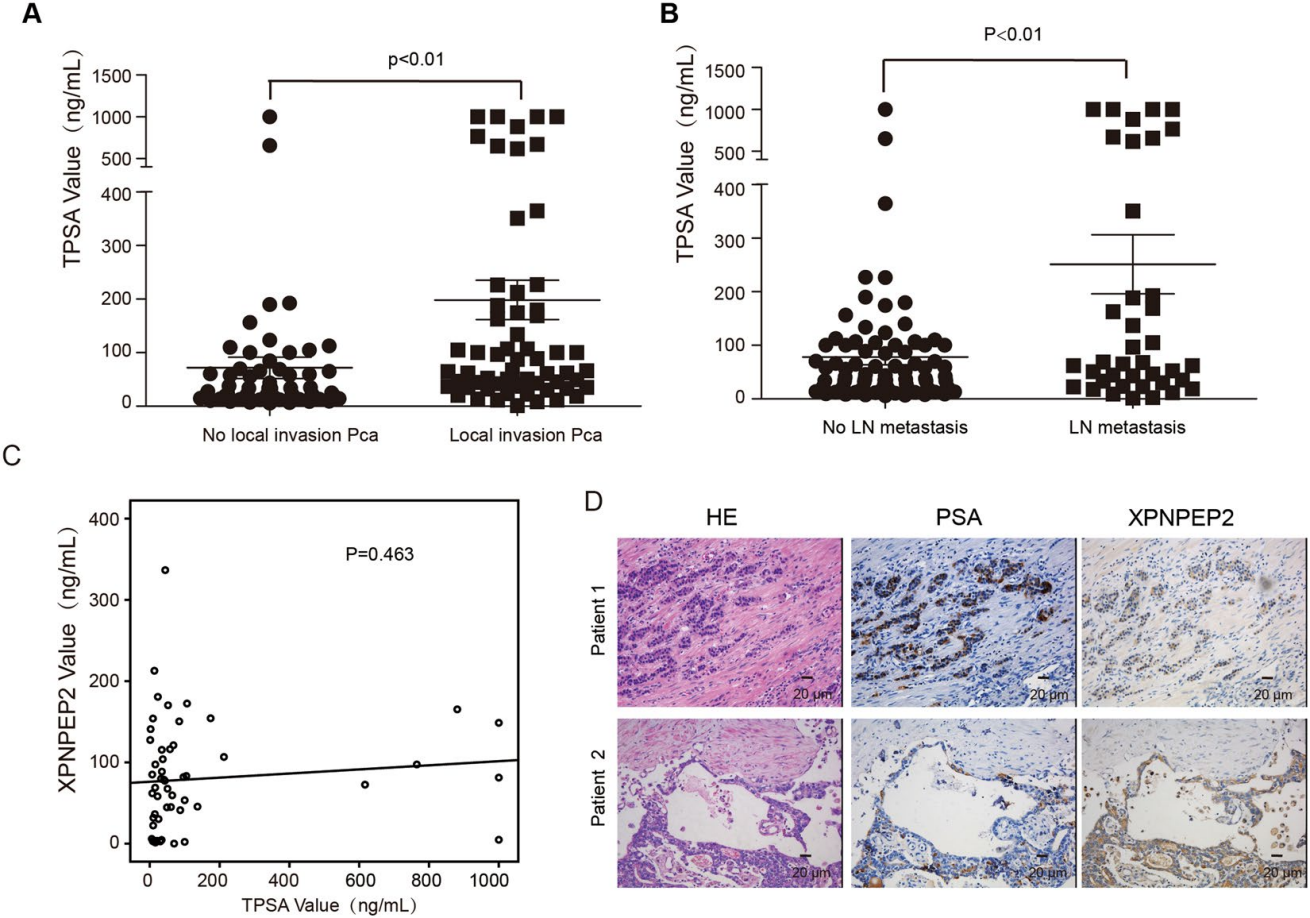
The correlation between PSA and XPNPEP2. The corresponding serum levels of PSA in Pca patients were analyzed (**A, B**), A correlation analysis between serum XPNPEP2 and PSA levels was performed (**C**). The XPNPEP2 and PSA were simultaneous stained by IHC in the serial sections, it is not in accord with the two gene expression in two representative Pca patients (**D**), scale bar, 20 um.

Western blot assays showed that the immunoreactivity bands with anti-XPNPEP2 was about 50 KD in serum samples (Fig. 3A). In contrast to its expression in tissues from Pca patients, serum XPNPEP2 levels were not significantly different when comparing Pca patients (77.35 ± 7.72 ng/mL), with normal (85.82 ± 8.16 ng/mL) and BPH patients (76.94 ± 12.86 ng/mL) (Fig. 3B). For further investigation, we analyzed Pca subtypes and found that the serum XPNPEP2 levels in patients with locally invasive Pca (95.91 ± 9.70 ng/mL) were higher than the levels in Pca patients without local invasion (77.58 ± 9.80 ng/mL) (*p* = 0.19) (Fig. 3C). Serum XPNPEP2 levels in Pca patients with LN metastasis (142.7 ± 14.40 ng/mL) were also significantly higher than levels in Pca patients without LN metastasis (61.63 ± 5.50 ng/mL) ( *p* < 0.01) (Fig. 3D). Meanwhile, serum XPNPEP2 values showed statistical significance between Pca patients with LN metastasis and healthy males or BPH patients (showed in Supplementary Fig. 3). The corresponding serum PSA levels of Pca patients were analyzed and also found to be associated with LN metastasis (Fig. 4A, B). In addition, we also observed a few Pca patients with outlier levels of PSA. When these outliers were excluded, the serum XPNPEP2 levels in Pca patients with LN metastasis were higher than levels in Pca patients without LN metastasis (Supplementary Fig. 4A, B).

### Correlation analysis

To explore whether there is an association between serum XPNPEP2 levels and PSA levels in Pca patients, a correlation analysis was performed. The result demonstrated that serum XPNPEP2 levels were independent of serum PSA levels (Fig. 4C). Similarly, the protein expression in Pca tissue as detected by IHC also revealed no relation between PSA and XPNPEP2. As shown in Fig. 4D, patient 1 had strong PSA but weak XPNPEP2 expression, oppositely patient 2 had weak and focal PSA but strong XPNPEP2 expression.

### ROC analysis of XPNPEP2 and PSA levels in Pca patients with LN metastasis

**Figure 5:**
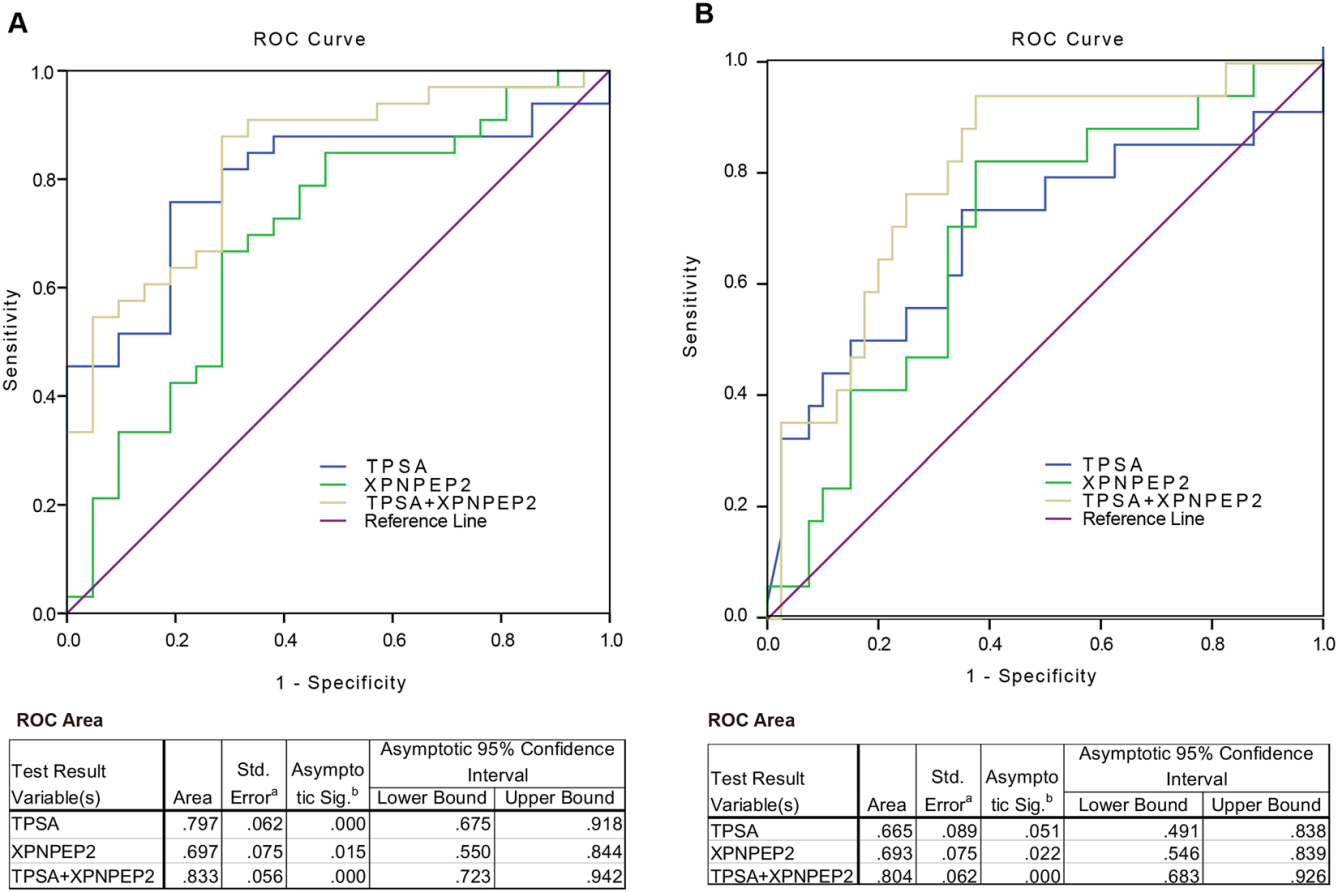
ROC curve tests of serum XPNPEP2 and PSA. ROC curves of XPNPEP2, PSA and both of them for patients with LN metastasis versus patients without (**A**). Outliers of PSA (>500 ng/ml) were excluded and ROC curves were generated for XPNPEP2, PSA and both of them for patients with LN metastasis versus patients without LN metastasis (**B**).

To validate the potential usefulness of serum XPNPEP2 as a noninvasive biomarker for predicting the risk of lymph node involvement in Pca patients, an Receiver operating characteristic (ROC) curve analysis was performed using serum XPNPEP2 levels alone or in combination with PSA. A comparison was made between Pca with LN metastasis and Pca without metastasis, which yielded area under the curve (AUC) value of 0.697, 0.797 and 0.833 for XPNPEP2, PSA and their combination, respectively (Fig. 5A). Therefore, combined application of serum XPNPEP2 levels and PSA levels might improve diagnostic accuracy of Pca patients with LN metastasis.

When the PSA outliers were excluded from the Pca patients with lymph node metastasis, the AUC for serum XPNPEP2 was 0.693, while for PSA decreased to 0.665. Nonetheless, AUC value of a combination of PSA and XPNPEP2 was 0.804 (Fig. 5B). These results indicated that, especially for patients with low PSA levels, the XPNPEP2 test may be useful for predicting lymph node metastasis when combined with the PSA test.

## Discussion

Aminopeptidase P (APP or XPNPEP2) is an aminoacylproline hydrolase widely distributed in prokaryotes and eukaryotes that specifically removes the N-terminal amino acid from peptides with a penultimate prolyl residue^11^. Several studies have demonstrated the correlation between polymorphisms and haplotype mutations of XPNPEP2 and ACEI-induced angioedema. There are fewer studies investigating the role of XPNPEP2 in cancer^13,16^. However, researches that reveal the role of XPNPEP2 in cancer are insufficient.

We confirmed that the expression of XPNPEP2 was significantly lower in prostate cancer tissues than normal prostate tissues. However, there was a discrepancy between the plasma and tissue levels of XPNPEP2 in Pca patients. Our study demonstrated that level of serum XPNPEP2 had correlation with lymph node metastasis. In addition, we analyzed XPNPEP2 gene alterations using data extracted from the cBioPortal online analysis tool and found that metastatic prostate cancer has the highest frequency of XPNPEP2 gene amplification^17,18^. Using a gene prioritization method (GP-MIDAS-VXEF) to compare prostate cancer and lymph node metastasis, Carlos Roberto Arias et.al demonstrated that XPNPEP2 is a metastatic gene candidate^19^. Additionally, Cheng te.al revealed that XPNPEP2 facilitated cervical cancer cell invasion and migration by inducing epithelial-mesenchymal transition^20^. Bioinformatic methods also verified that serum XPNPEP2 could be a potential biomarker for lymph node metastasis.

Lymph node metastasis is a powerful prognostic factor in guiding clinical decision-making in terms of surgery, follow-up scheduling and adjuvant therapies. Several previous studies created a nomogram to predict the risk of lymph node involvement (LNI)^21–23^. The three-variable nomogram includes basic grade clinical variables (pretreatment PSA, clinical stage, and biopsy Gleason grade). In this study, we also verified that PSA levels correlated with Pca aggressiveness and tumor volume. However, approximately 5–10% of prostatic adenocarcinomas produce little PSA, and some patients with low PSA levels have higher rates of adverse pathologic features^4^. Especially for patients with low PSA levels, the XPNPEP2 test may be useful for predicting lymph node metastasis when combined with the PSA test. The expression of PSA is mainly induced by androgens. And Christian Drouet et.al unearthed that patients with HAE receiving androgen prophylaxis showed a significantly higher plasma APP activity. Androgen can regulate the PSA expression and XPNPEP2 activity, but mechanisms are different. PSA expression was mainly regulated by the androgen receptor (AR) at the transcriptional level. The author hypothesized that the effect that androgens have on the circulating APP activity results from a potentiating effect on a glycosylphosphatidyli-nositol hydrolase activity. This includes metalloprotease and phospholipase mechanisms, with subsequent release of glycosylphosphatidylinositol-anchored proteins, which are responsible for the shedding of APP from the endothelial membrane. May be that is why BPH tissues have higher XPNPEP2 expression than PCa tissues which are not advanced^24^.

The major limitation of this study is the relatively small serum sample pool from Pca patients with lymph node metastasis. We believe that larger studies are required to confirm our findings. In addition, this was a single institution study, and a large scale multicenter and multiracial study will be needed to evaluate the value of XPNPEP2 as a lymph node metastatic biomarker for Pca.

In summary, we demonstrated that serum XPNPEP2 level was associated with lymph node metastasis and local invasion. Combining PSA and XPNPEP2 can increase the efficiency of predicting LN metastasis compared to the efficiency of using PSA or XPNPEP2 alone. Especially for Pca patients with low serum PSA levels, XPNPEP2 was a powerful marker for predicting LN metastasis.

## Materials and Methods

### Serum collection

Between December 2014 and March 2017, 128 serum samples of prostate cancer patients, 70 samples of healthy males and 40 samples from patients with prostate hyperplasia were obtained from the clinical laboratory of Tongji hospital (Wuhan, China). All serum samples were collected prior to radical prostatectomy and chemotherapy. Patient clinical data were recorded, including their age, Gleason score, histopathological findings, PSA levels, positive lymph nodes and medication history. Ethical approval was obtained from the ethical management committees of Tongji hospital according to the guidelines of Helsinki conventions (TJ-IRB20180401). All participants provided written informed consent before being sampled for our study.

### Tissue microarrays and prostate cancer tissue

High-density multiple organ tumor tissue array (MC5003a, US Biomax, Inc.) was used for the initial screening of XPNPEP2 expression. This 500 core tissue microarray includesd the 20- most common types of cancer (25 cases/type) and normal controls (5 cases/type) with TNM classifications and pathological grades of the tumors. On the base of this screening, XPNPEP2 expression was further analyzed in individual organs, using the prostate cancer tissue microarray PR1921 which contains adjacent normal prostate tissues and normal tissues as controls. This array included the TNM, clinical stage and pathological grades and the level of IHC markers (PSA) from 96 cases arrayed in duplicate to yield 192 cores. To analyze XPNPEP2 expression associated with invasion and metastasis, 90 formalin-fixed paraffin-embedded (FFPE) prostate cancer tissue samples, including 30 from patients with localized Pca, 30 from patients with local invasion, 30 from patients with lymph node metastasis and corresponding adjacent normal prostate tissues were collected from the pathology department of Tongji hospital (Wuhan, China). The pathologic features were determined by urological pathologists using standardized protocols.

### XPNPEP2 expression by immunohistochemistry

The above tissue microarrays and prostate cancer tissue samples were subjected to immunohistochemistry with rabbit anti-XPNPEP2 (1:250, GTX109995, GeneTex, USA) and anti-PSA antibodies (1:250, ab76113, Abcam, USA). Briefly, FFPE sections were deparaffinized in xylene and then rehydrated with descending concentrations of alcohol. Antigen retrieval was performed by microwave heating in citric acid buffer (pH 6.0) for 12 min at 100 °C followed by cooling at room temperature. Endogenous peroxidase was blocked by 3% hydrogen peroxide for 30 min at 37 °C. Then, the samples were incubated with a primary antibody overnight at 4 °C. The immunohistocemistry procedure was continued using the standard protocol from a goat anti-rabbit IHC kit (SP-9001; ZSGB-BIO). Chromogen was developed with a DAB chromogenic substrate kit (GoodBio, Wuhan). Then, sections were counterstained with hematoxylin. Immunostaining was evaluated and a blind score assigned by two experienced pathologists with no prior knowledge of the pathological parameters. The immunoreactivity scoring (IRS) system was based on the staining intensity on a scale of 0 to 3 (0 = negative; 1 = weakly positive; 2 = moderately positive; 3 = strongly positive).

### GEPIA dataset analysis

Gene Expression Profiling Interactive Analysis (GEPIA) is an interactive web server for estimating mRNA expression data based on 9,736 tumors and 8,587 normal samples in the Cancer Genome Atlas (TCGA) and Genotype-tissue Expression dataset projects. GEPIA was employed for mRNA expression analysis of XPNPEP2 in prostate cancers tissues and normal ones^25^.

### Western blot assay

To analyze the serum XPNPEP2, all serum was diluted 1:5 in lysis buffer. Each sample was loaded in SDS-acrylamide gels and transferred on Immobilon PVDF membranes, the transferred membranes were incubated overnight with an appropriate dilution of the primary anti-body at 4 °C (anti-XPNPEP2, 1:1000, GTX109995, GeneTex, USA), the horse-radish peroxidase-conjugated anti-rabbit or anti-mouse IgG using as the secondary antibody was diluted at 1:1000. The immune complexes for western blot were visualized via fluorography using an enhanced ECL system (Thermo Fisher Scientific, San Jose, CA, USA).

### ELISA assay

Serum levels of XPNPEP2 were measured by using a human XPNPEP2 ELISA pair set (cat: SEK11903, Sino Biological, Beijing). The ELISA assay was performed as follows. A 96-well microplate was coated with 100 μL monoclonal antibody specific for XPNPEP2 (2 μg/mL) overnight at 4 °C. The wells were then washed three times and blocked, and 100 μL of standards or samples (diluted 1:200) were added to individual wells. Each well was aspirated and washed three times with at least 300 μl of buffer per wash. Subsequently, 100 μL of HRP-conjugated anti-XPNPEP2 monoclonal antibody (0.5 μg/mL) was added. The aspiration and wash cycle was repeated, and then 100 μL of TMB substrate solution was loaded. 50 μL of stop solution was added and the absorbance of each well at 450 nm was measured. XPNPEP2 concentrations were calculated based on the curve generated from the standards.

### Statistical analysis

Between-group differences for continuous variables with a normal distribution were tested with Student’s t test. The Mann-Whitney U test was employed to compare continuous variables that did not have a normal distribution. Receiver operating characteristic (ROC) curves were constructed to determine the diagnostic performance of serum XPNPEP2 levels and PSA levels in distinguishing prostate cancer patients with localized invasion or lymph node metastasis from patients with low-grade prostate cancer. The area under the ROC curve (AUC) was used to assess the predictive power. Analyses were performed using Prism 5 software (GraphPad) and SPSS 13.0. Two-sided P values of <0.05 were considered statistically significant.

## Supplementary information

supplementary information
