# Supplementary material for: XPNPEP2 is associated with lymph node metastasis in prostate cancer patients
Source: Sci Rep. 2019 Jul 11;9:10078. doi: 10.1038/s41598-019-45245-5 (PMC6624198; doi:10.1038/s41598-019-45245-5)

**XPNPEP2 is associated with lymph node metastasis in Pca patients**

Fei Li^a,#^,Yun Dai^a,#^, Hao Xu^b^,Kecheng Huang^a^Ying Zhou^a^,Danfeng Luo^a^, Ding Ma^a^, Ling Xi ^a^, Mengqin Lv^a,c,*^,Xiangyi Ma ^a,*^

**Supplementary figure legends**

Supplementary Figure 1. The XPNPEP2 expression overview by IHC in various normal organs. XPNPEP2 was strongly positive in the renal proximal tubule, moderately positive in prostate tissues, weakly positive in the liver, intestine and pancreatic islets and negative in the lung.

Supplementary Figure 2. GEPIA was employed for mRNA expression analysis of XPNPEP2 in prostate cancers and healthy males.

Supplementary Figure-3. Comparison of the XPNPEP2 levels in healthy males, BPH patients and patients diagnosed with LN-metastatic PCa.

Supplementary Figure-4. When PSA outliers were excluded, serum XPNPEP2 levels in Pca patients with different clinicopathologies. Outliers of PSA (> 500 ng/ml) were excluded, C, Plots illustrating the serum XPNPEP2 levels in Pca patients with local invasion versus without local invasion. D, Plots showing the serum XPNPEP2 levels in Pca patients with LN metastasis versus without LN metastasis.

**Supplementary Figures:**

Supplementary Figure1. The XPNPEP2 expression overview by IHC in various normal organs. XPNPEP2 expression was strongly positive in the renal proximal tubule, moderately positive in prostate tissues, weakly positive in the liver, intestine and pancreatic islets and negative in the lung.


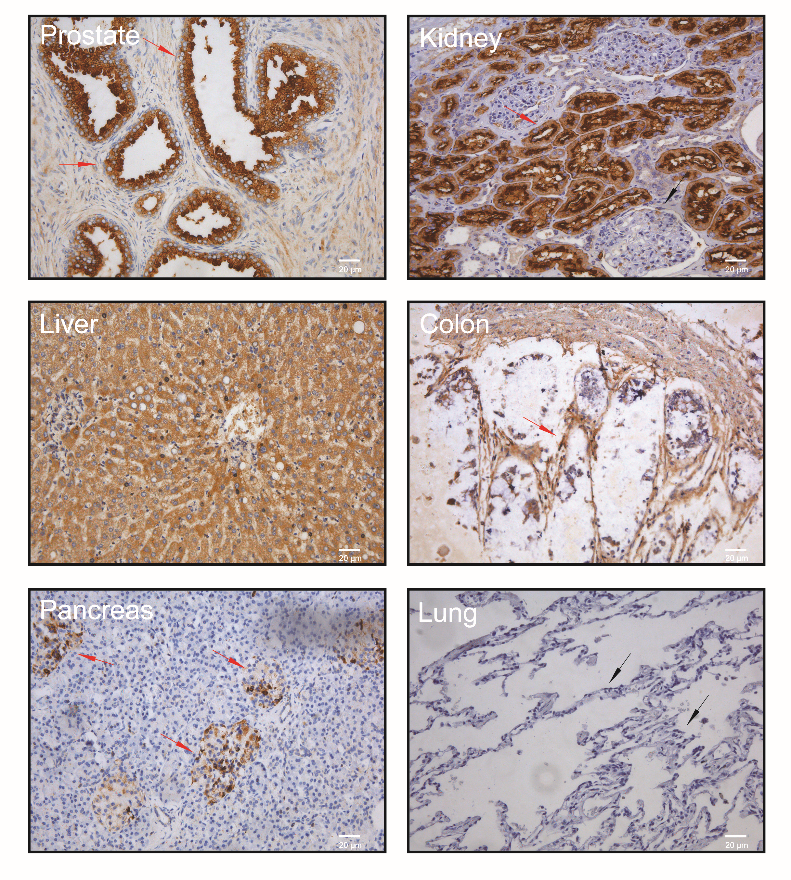


Supplementary Figure-2. GEPIA was employed for mRNA expression analysis of XPNPEP2 in prostate cancers and healthy males..


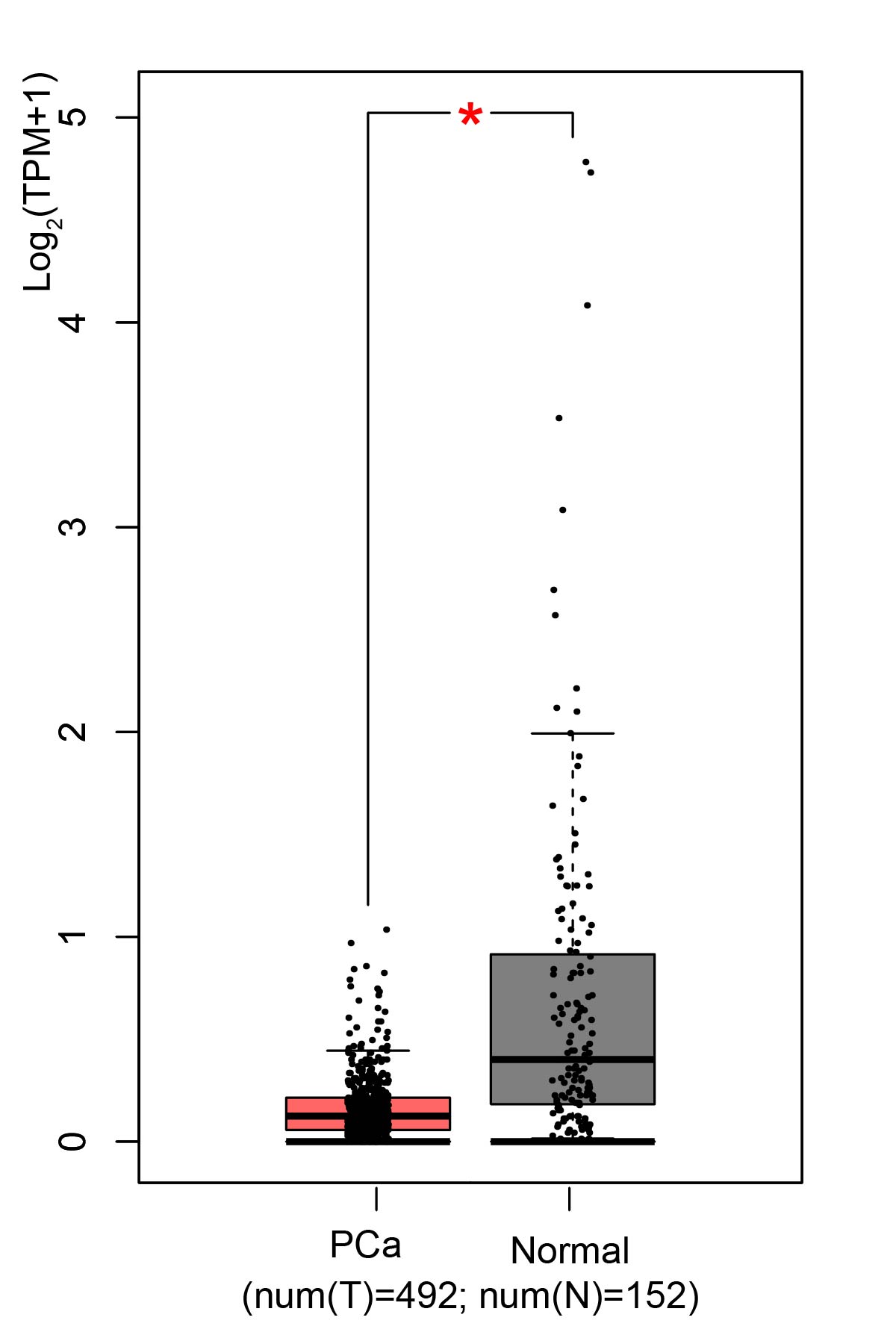


Supplementary Figure-3. Comparison of the XPNPEP2 levels in healthy males, BPH patients and patients diagnosed with LN-metastatic PCa.


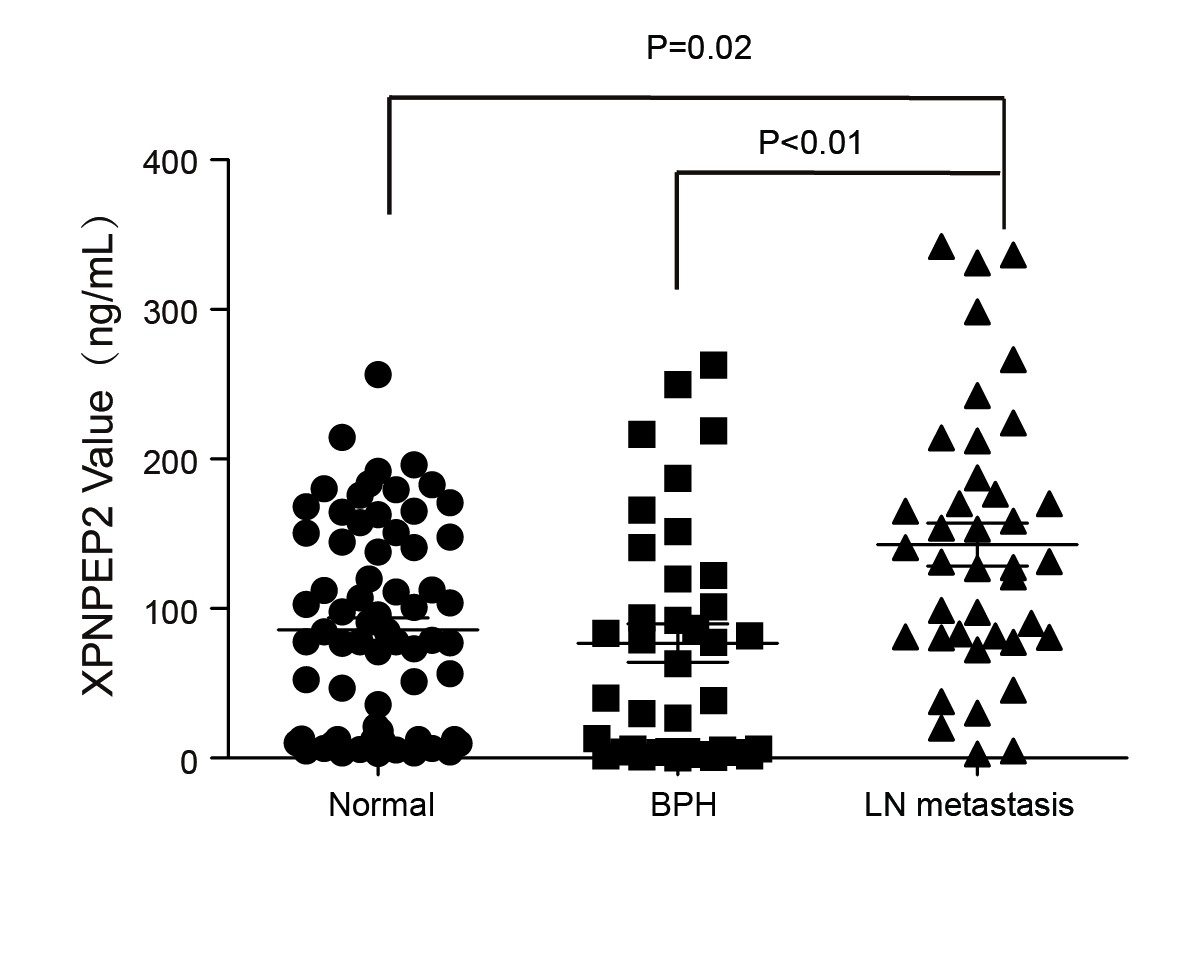


Supplementary Figure-4. When PSA outliers were excluded, serum XPNPEP2 levels in Pca patient with different clinicopathologies. Outliers of PSA (> 500 ng/ml) were excluded, C, Plots illustrating the serum XPNPEP2 levels in Pca patients with local invasion versus without local invasion. D, Plots showing the serum XPNPEP2 levels in Pca patients with LN metastasis versus without LN metastasis.


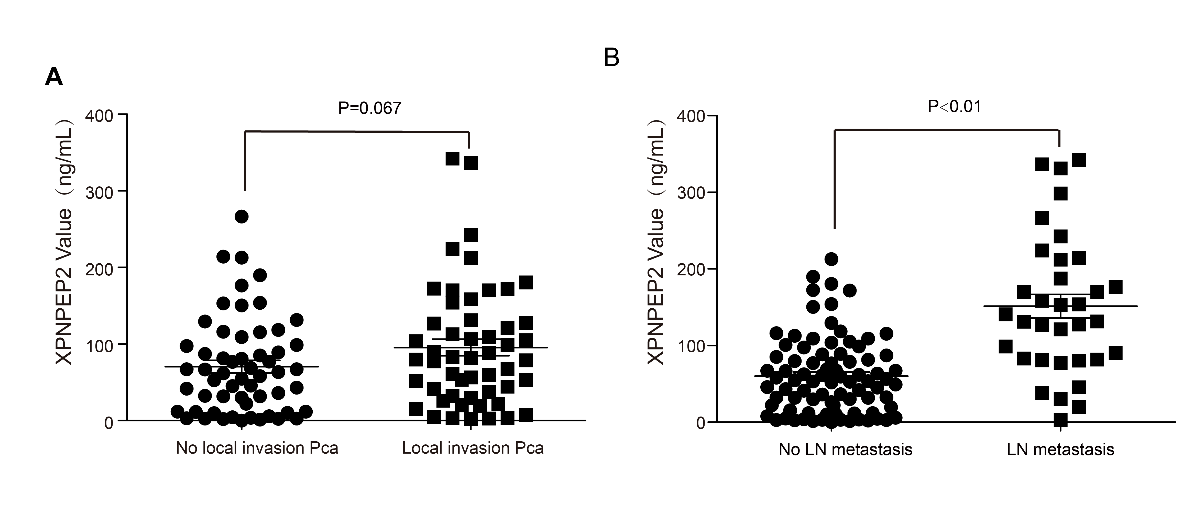

Supplement: Supplementary file 1 — supplementary information [file 41598_2019_45245_MOESM1_ESM.doc]
